# Minimising Risks of Reduced Genetic Diversity in Marine Restoration

**DOI:** 10.1111/eva.70257

**Published:** 2026-05-20

**Authors:** G. Wood, K. Filbee‐Dexter, T. Wernberg, M. A. Coleman

**Affiliations:** ^1^ UWA Oceans Institute and School of Biological Sciences University of Western Australia Crawley Western Australia Australia; ^2^ College of Science and Engineering, Flinders University Adelaide South Australia Australia; ^3^ Institute of Marine Research, Flødevigen Research Station His Norway; ^4^ NSW Department of Primary Industries and Regional Development, Fisheries New South Wales Australia

**Keywords:** adaptation, aquaculture, captive breeding, domestication, genetic management, genetics, marine restoration, risk

## Abstract

Marine habitat restoration is expanding globally and increasingly relies on aquaculture and hatchery‐based propagation, yet the genetic consequences of producing and outplanting large numbers of habitat‐forming organisms remain poorly evaluated. A lack of best‐practice guidelines and insufficient genetic monitoring creates risks including bottlenecks, inbreeding and domestication, with long‐term impacts possible for both restored and remnant populations. Here, we assessed the impacts of hatchery processes on genetic diversity and domestication in the kelp, *Ecklonia radiata*, by genotyping wild donor populations and their hatchery‐bred *F*_1_ cohorts. Despite using ~100 donor adults per site, hatchery‐bred *F*_1_ individuals showed clear reductions in genetic diversity. On average, neutral heterozygosity declined by ~20%–30%, and more than half of the percentage of polymorphic loci was lost, indicating strong genetic drift and erosion of potentially locally adapted variation. Moreover, a small number of outlier loci were strongly associated with hatchery conditions, indicating early selection and domestication responses within a single generation. *F*_1_ cohorts also exhibited elevated inbreeding and sharp reductions in effective population size (*N*_e_ falling from ~500 in the wild to as low as 21 in the hatchery). These findings demonstrate that unmonitored propagation can rapidly alter the genetic composition of foundation species under hatchery conditions, potentially undermining adaptive capacity and field performance. Incorporating genetic guidelines and monitoring into restoration programs will be essential to support long‐term persistence and evolutionary resilience.

## 1 Introduction

Marine ecosystems are experiencing unprecedented degradation due to human impacts and accelerating climate change (Halpern et al. 2008; Doney et al. 2012; IPCC 2023). Nearly half of the world's foundational coastal habitats, including coral reefs, kelp forests, seagrasses and saltmarshes, have been lost or severely degraded (Wernberg et al. 2024). In response, global initiatives such as the UN Decade on Ecosystem Restoration and the Kunming–Montreal Global Biodiversity Framework call for rapid scaling of restoration actions to 2030 (UN 2019; Duarte et al. 2020; CBD 2021).

To meet these restoration goals at scale, marine practitioners are increasingly turning to aquaculture propagation in hatcheries. This mirrors terrestrial nurseries that supply vast quantities of seed and builds on decades of experience in fish and shellfish stock enhancement (Naish et al. 2007; Kitada 2018). Aquaculture is now central to large‐scale oyster and mussel restoration (Carranza and zu Ermgassen 2020), salt marsh and mangrove restoration (Vanderklift et al. 2020), and coral initiatives that outplant hundreds of thousands of fragments and juveniles annually (Lirman and Schopmeyer 2016; Boström‐Einarsson et al. 2020). Comparable approaches are rapidly emerging for macroalgae, particularly kelps, with new programs developing across > 17 ecoregions globally (Wood et al. 2024).

Genetic diversity underpins resilience and adaptive capacity in natural populations, shaping responses to environmental change across ecological and evolutionary scales. As a result, it is a foundational pillar of ecological restoration (IUCN 2013; Gann et al. 2019). In practice, the benefits of genetic diversity depend on how it is distributed among donor source populations and whether locally adapted variation is retained, as the provenance of donor stock strongly influences immediate outplant performance. Poorly matched genotypes may show reduced survival, growth and recruitment when introduced into contrasting thermal, hydrodynamic, or biotic regimes (Leimu and Fischer 2008; Hereford 2009). This is of particular concern for habitat‐forming, sessile taxa where local adaptation is common (Hays et al. 2021; Veenhof et al. 2023) and associated pressures such as temperature and grazing can also cause high restoration failure rates (Eger et al. 2022; Wood et al. 2024; Kendrick et al. 2025). High levels of genetic variation supported by adequate effective population size can also reduce the risk of genetic drift and inbreeding, maintain fecundity and mating success, and broaden tolerance to environmental and biotic stress, thereby sustaining establishment, self‐recruitment and multi‐generation persistence (Sgrò et al. 2011; Weeks et al. 2011; Wernberg et al. 2018). Genetic diversity also often underpins functional traits such as growth, morphology, nutritional or carbon content that underpin key ecosystem functions (Crutsinger et al. 2006; Whitham et al. 2006; Starko et al. 2026) that are important to maintain in restoration programs.

The risks of inadequate genetic management causing reduced genetic diversity during hatchery processes have been demonstrated in decades of captive breeding and stock enhancement research. Across finfish, shellfish and terrestrial plants, hatchery rearing often reduces effective population size, increases inbreeding and selects for traits favoured under benign culture conditions rather than in the wild (Hamilton 1994; Araki et al. 2007; Fraser 2008). Genomic changes can arise in a single generation (Christie et al. 2012, 2014, 2016), while fitness declines often emerge after several generations as genetic load accumulates. Poorly designed releases can also compromise genetic diversity in the wild, for example by reducing system‐wide *N*_e_ via the Ryman–Laikre effect (Ryman and Laikre 1991; Laikre et al. 2010).

In fisheries, these risks are generally mitigated through broodstock guidelines, rare‐allele retention modelling and genetic monitoring of *N*_e_ and diversity (Camara and Vadopalas 2009; FAO 2017). Equivalent conservation genetics frameworks, however, are almost entirely absent for marine habitat‐forming taxa such as kelps and corals, despite rapid declines in these ecosystems and growing investment in restoration (Eger et al. 2023; Wernberg et al. 2024; Wood et al. 2024).

Kelp forests dominate temperate reefs worldwide but have suffered widespread decline due to warming, marine heatwaves, eutrophication and overgrazing (Krumhansl et al. 2016; Eger et al. 2024). Hatchery‐based kelp restoration programs are now emerging globally (e.g., Wood et al. 2024), yet very few monitor genetic outcomes (Wood et al. 2020; Dykman et al. 2025), hindering the development of best‐practice guidelines to avoid unintended genetic risks. These same challenges apply to the design of macroalgal biobanks, where stored material must typically pass through nursery or hatchery phases prior to outplanting. Critical knowledge gaps include how to select optimal donor numbers, optimal breeding designs and the risks of domestication during hatchery rearing.

Here, we use kelp forests as a case study to empirically assess the genetic risks associated with hatchery reared restoration activities. We genotyped wild donors from two environmentally distinct populations and their hatchery‐bred *F*_1_ cohorts to test if propagation processes impacted genetic diversity in one generation. Specifically, we asked whether our breeding protocols impacted genetic diversity at neutral and putatively adaptive outlier loci, increased inbreeding, reduced effective population size, or produced signals of selection to hatchery conditions (domestication). We then coupled these data with forward genetic simulations to quantify how donor number influences diversity retention across outplanted generations, providing quantitative targets for hatchery processes and a practical genetic monitoring framework for kelp restoration programs and policy.

## 2 Methods

### 2.1 Study Species

*Ecklonia radiata* dominates temperate reefs across southern Australasia and southeast Africa (Wernberg et al. 2019). Like other Laminariales, it has a haplodiplontic life cycle, with microscopic gametophytes and macroscopic sporophytes, enabling both vegetative propagation and zoospore‐based seeding. The species has experienced widespread climate‐ and water‐quality‐driven declines across Australia (Connell et al. 2008; Vergés et al. 2016; Wernberg et al. 2016; Young et al. 2023).

### 2.2 Restoration Hatchery Experiment

We sampled two genetically and environmentally distinct Western Australian kelp populations: Port Gregory (“warm”) and Perth (“cool”) (Vranken et al. 2021; Minne 2024). During peak reproduction (autumn 2022), ~100 reproductively active adults per site were collected by SCUBA. Individuals were collected at ~12 m depth from exposed rocky reef habitats at each site. To minimise sampling of closely related individuals, thalli spaced approximately 1–2 m apart were haphazardly selected across an area of ~100 m^2^. Samples were transported on ice in insulated containers to the UWA Watermans Bay Marine Science Centre and processed within 8 h.

**FIGURE 1:**
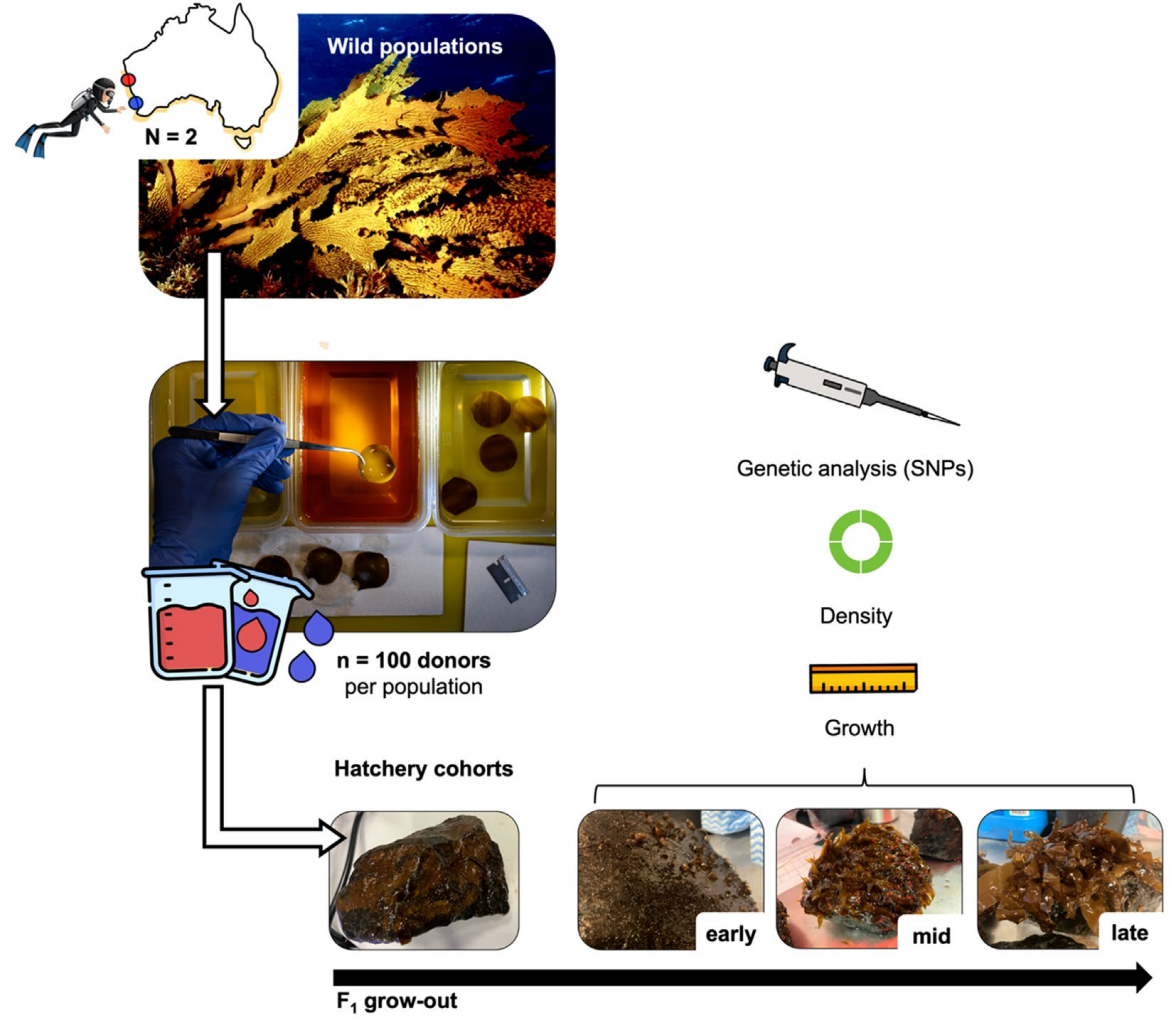
Experimental design showing collection of warm and cool donors (*n* = 100 each), mass spawning, hatchery culture, and temporal sampling (5, 7, and 9 months post‐spore release) of *F*_1_ cohorts.

For each population, we pooled sorus tissue from 100 of the donors from each site to produce two mass‐spawned hatchery cohorts. Two 3 cm sorus discs per donor were excised, hydrated in filtered seawater, and gently agitated to release zoospores. Wild donor tissue was sampled for genetic analysis at this time following the protocol described below (Figure 1). Zoospore suspensions were standardised (~5000 mL^−1^) and used within 24 h to seed basalt substrates (12 rocks per tank; 8 tanks per provenance) in 50 × 50 cm culture tanks containing UV‐treated seawater. Seasonal temperature and light regimes followed ambient conditions, and flow‐through seawater commenced after 2 weeks.

*F*_1_ sporophytes were destructively sampled at 5, 7 and 9 months post‐seeding by selecting one sporophyte from three different rocks per tank. Tissue was rinsed, snap‐frozen, freeze‐dried and sent to Diversity Arrays Technology (Canberra, Australia) for DNA extraction and DArTseq genotyping (Kilian et al. 2012). At the 7‐month time point, the warm treatment was sampled twice (six days apart), resulting in an increased sample size at this time point. *F*_1_ samples were pooled for downstream analyses of genetic selection and structure; however, genetic diversity measures were calculated for each time point to determine if there was an impact of time. Genetic diversity metrics comparing the two sampling events in month 7 are presented separately in the Supporting Information (Table S1).

### 2.3 Genetic Analyses

Genetic analyses were conducted in R 4.3.1 (R Core Team 2023). Of 49,220 initial SNPs (mean depth = 6.79; 18.3% missing data), filtering was conducted sequentially in dartR (Gruber et al. 2018; Mijangos et al. 2022) by retaining loci with read depth between 5 and 12, reproducibility ≥ 0.96 and removing secondary loci at random. Loci with minor allele frequency ≤ 1% were excluded, followed by filtering for missingness (retaining loci with < 90% missing data and individuals with < 80% missing data), and removal of monomorphic loci. This resulted in a final dataset of 1601 loci across 301 individuals.

We identified outlier loci using redundancy analysis (RDA), testing associations with (i) provenance (“warm” vs. “cool”) and (ii) hatchery environment (wild vs. hatchery) (Rellstab et al. 2015). RDA is a multivariate ordination method that identifies associations between loci and explanatory variables, capturing putative multilocus responses to selection (Forester et al. 2018; Capblancq and Forester 2021). Predictor variables (population and hatchery versus wild cohorts) were coded as binary dummy variables. Redundancy Analysis (RDA) detected significant associations between 42 unique loci and the fitted variables (see Results). Candidate loci were annotated via BLASTx (e‐value < 10^−1^) against the closest gene model *Ectocarpus siliculosus* (v2) from the ORCAE database (https://bioinformatics.psb.ugent.be/gdb/ectocarpusV2/). Subsequent analyses were conducted using neutral, provenance‐associated, and hatchery‐associated subsets.

Population structure was visualised by PCoA (gl.pcoa, dartR) using bitwise distances. Pairwise genetic differentiation (Weir & Cockerham *F*_ST_) was estimated with 999 bootstrap replicates and FDR‐corrected *p*‐values.

We assessed donor representation in the *F*_1_ cohorts using the neutral dataset. Effective population size (*N*_e_) was estimated using the molecular coancestry method (Nomura 2008) implemented in NeEstimator v2.1 (Do et al. 2014) via gl.LDNe (dartR). Genetic diversity metrics included observed and expected heterozygosity (*H*_o_, *H*_e_), percentage of polymorphic loci (PL), rarefied allelic richness (AR), private alleles (PA) and inbreeding (*F*_IS_). *F*_IS_ estimates were assessed for significance using 1000 bootstrap replicates across loci to generate 95% confidence intervals in hierfstat (Goudet and Jombart 2024). These metrics are generally robust to moderate differences in sample size; allelic richness was rarefied and effective population size estimates incorporate sample size corrections, although we note that the proportion of polymorphic loci may be more sensitive to sample size and should be interpreted with caution when comparing donor and *F*_1_ cohorts. Metrics were calculated separately for neutral and outlier loci using dartR, hierfstat and poppr (Kamvar et al. 2014).

We also explored how the number of restoration donors used might influence genetic diversity in future restored populations. Neutral genetic patterns were simulated for the *F*_1_–*F*_100_ offspring cohorts using gl.sim.offspring in dartR, utilising a 1:1 sex ratio and random mating (Praeger et al. 2022) and assuming no mixing with any remaining extant kelp plants in restored sites. Initial donor numbers ranged from two to 100 (increments of two up to 10, then by 10 hereafter), each bootstrapped 100 times, with 500 randomly mating offspring for each of 100 generations. Diversity (*H*_e_, PL) was calculated for *F*_1_, *F*_3_, *F*_10_ and *F*_100_ using gl.report.heterozygosity.

### 2.4 Survival and Growth

Kelp density and size on rocks in the hatchery were quantified at 5 and 7 months postseeding. Density was measured from three 1 cm‐radius quadrats per rock (3 rocks per tank). Linear mixed models (LMMs) were fitted using lme4 (Bates et al. 2015), with provenance (“warm” vs. “cool”) and time as fixed effects and tank nested within provenance as a random effect. Significance was assessed using Type III ANOVA (Kenward–Roger df). Estimated marginal means were obtained using emmeans (Lenth and Piaskowski 2025). Model assumptions were checked via residual diagnostics.

## 3 Results

### 3.1 Genetic Analyses

Redundancy Analysis (RDA) detected significant associations between 42 unique loci and the fitted variables. Of these, 27 loci (1.7% of all SNPs) were associated with provenance and 15 loci (0.9%) were associated with hatchery cultivation (Figure S1). BLAST analysis revealed that none of the candidate loci under hatchery selection had matches to annotated genes in the *Ectocarpus* genome. However, one locus (46357600‐33), polymorphic only in the warm donor population, was linked to growth, immunity and membrane transport. Two loci with polymorphic alleles exclusive to the cool donor population (100215211‐30 and 54904122‐13) were associated with protein transport and RNA processing.

Linkage disequilibrium analysis indicated that three‐quarters of Cool donors contributed to the *F*_1_ hatchery gene pool (estimated *N*_e_ = 74, Table 1), while warm hatchery kelp had an *N*_e_ of just 21, about a fifth of the number of donors used (Table 2). In both cases, effective population sizes were substantially lower (7–25 X) than in the wild populations (cool *N*_e_ = 512 and warm *N*_e_ = 495).

**TABLE 1:** Estimates of effective population size (*N*_e_) for wild and hatchery‐cultivated cohorts of *Ecklonia radiata* of warm and cool provenance calculated with *N*_e_ Estimator.

	*N*_e_^a^	Overall *R* ^2^	Expected *R* ^2^	*N* donors (%)	*n*
Cool donor	512 (473, 557)	0.011	0.011		100
Warm donor	495 (441, 563)	0.012	0.011		100
Cool hatchery	74 (68, 80)	0.034	0.033	100 (74%)	36
Warm Hatchery	21 (21, 21)	0.032	0.018	100 (21%)	62

*Note:* Calculated with 1559 neutral loci using the linkage disequilibrium method. 95% confidence intervals shown in brackets. *N* donors = number of donor kelp plants and the % that contributed to the *F*_1_ generation. *n* = number samples used in analysis.

^a^ Lower and upper CI shown in brackets.

**TABLE 2:** Genetic diversity metrics across time.

Population	Cohort	Age (months)	*n*	Neutral loci						Provenance‐associated outliers						Hatchery‐associated outliers					
				PL (%)	AR	PA (%)	*H*_o_	*H*_e_	*F*_IS_	PL (%)	AR	PA (%)	*H*_o_	*H*_e_	*F*_IS_	PL (%)	AR	PA (%)	*H*_o_	*H*_e_	*F*_IS_
***Warm***	***Wild***	**> 12**	**98**	**36.626**	**1.038**	**20.975**	**0.005**	**0.006**	**0.200**	**62.963**	**1.369**	**3.704**	**0.080**	**0.095**	**0.146**	**60.000**	**1.074**	**0.000**	**0.011**	**0.011**	**−0.005**
	***Hatchery***		**62**	**17.960**		**3.784**	**0.004**	**0.006**	**0.183**	**66.667**		**0.000**	**0.051**	**0.640**	**0.150**	**93.333**		**20.000**	**0.127**	**0.171**	**0.323**
		5	12	29.787	1.026	0.449	0.003	0.005	0.250	18.519	1.153	0.000	0.045	0.044	−0.003	73.333	1.670	0.000	0.130	0.221	0.360
		7	33	35.106	1.029	1.860	0.004	0.005	0.174	66.667	1.321	0.000	0.061	0.080	0.153	86.667	1.645	0.000	0.129	0.144	0.121
		9	17	38.830	1.037	1.155	0.004	0.006	0.366	29.630	1.224	0.000	0.037	0.059	0.245	80.000	1.658	0.000	0.112	0.156	0.248
***Cool***	***Wild***	**> 12**	**101**	**54.586**	**1.090**	**26.491**	**0.014**	**0.017**	**0.175**	**70.370**	**1.408**	**0.000**	**0.089**	**0.103**	**0.121**	**20.000**	**1.027**	**0.000**	**0.004**	**0.004**	**−0.005**
	***Hatchery***		**36**	**27.646**		**3.784**	**0.011**	**0.016**	**0.206**	**66.667**		**0.000**	**0.064**	**0.111**	**0.415**	**33.333**		**0.000**	**0.008**	**0.028**	**0.526**
		5	9	12.957	1.099	1.347	0.015	0.019	0.226	48.148	1.459	0.000	0.097	0.122	0.200	13.333	1.131	0.000	0.008	0.033	0.807
		7	18	16.164	1.078	1.411	0.009	0.015	0.361	55.556	1.428	0.000	0.054	0.093	0.348	6.667	1.063	0.000	0.004	0.017	0.773
		9	9	7.633	1.090	1.090	0.008	0.012	0.315	51.852	1.466	0.000	0.051	0.112	0.440	26.667	1.232	0.000	0.015	0.040	0.500

*Note:* Based on putatively neutral (*n* = 1559) and outlier loci associated with Provenance (*n* = 27) and Hatchery (*n* = 15), sequenced across Donor (*n* = 199) and Hatchery (*n* = 102) *Ecklonia radiata* cohorts. All *F*_IS_ estimates were significantly different from zero across groups, indicating consistent departures from random mating. The colour shading and bold formatting indicates these are the overall genetic diversity metrics calculated on each hatchery/wild cohort. In contrast, the rows below that are not coloured or in bold correspond to the hatchery cohorts broken down by age (5,7,9 months as indicated). Red corresponds to the warm population, blue corresponds to the cool population.

Abbreviations: AR = rarefied allelic richness; *F*_IS_ = inbreeding coefficient; *H*_e_ = expected heterozygosity; *H*_o_ = observed heterozygosity; PA = private alleles; PL = polymorphic loci.

**FIGURE 2:**
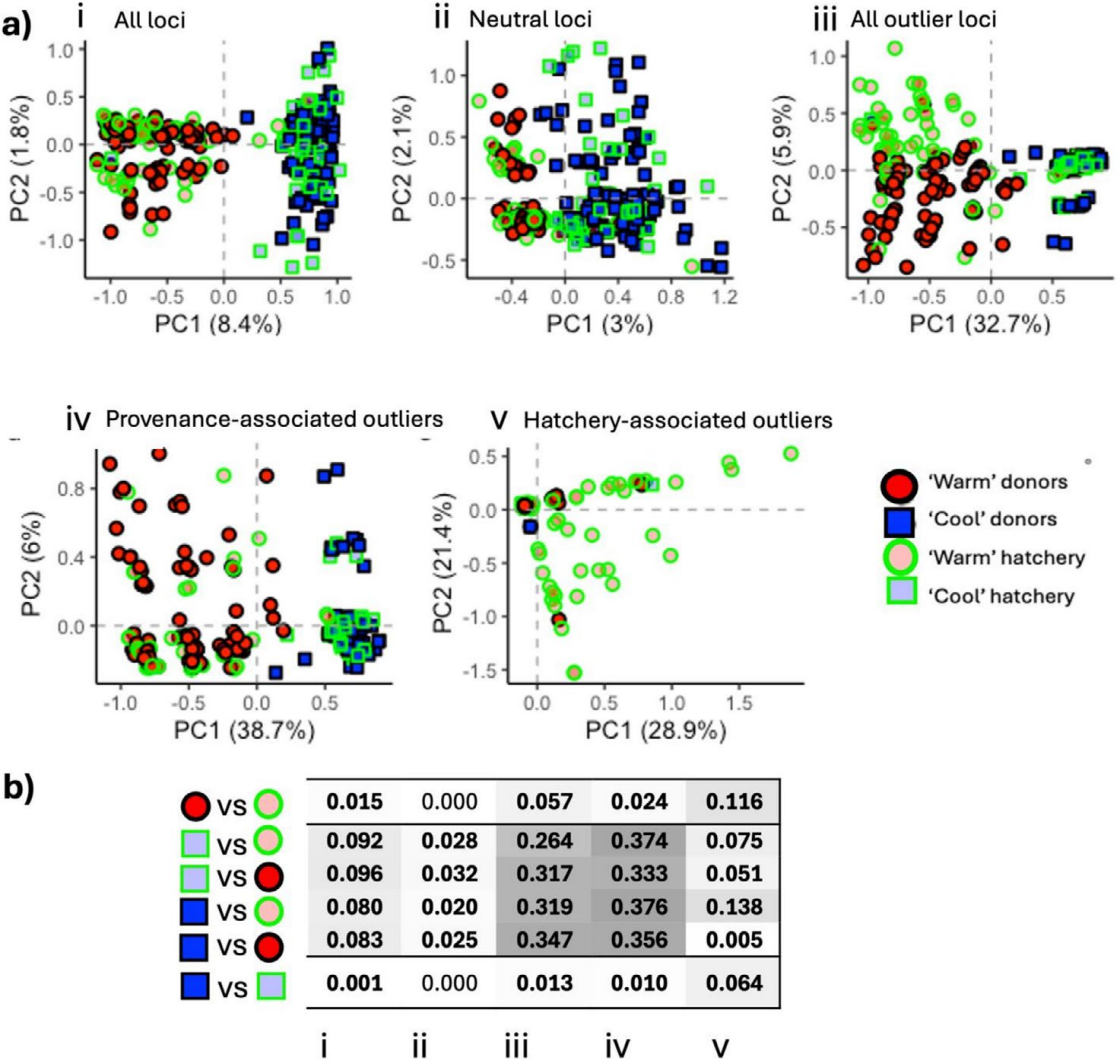
Genetic structure of donor kelp and hatchery‐cultivated cohorts. (a) Constrained ordination of axis 1 and 2 from Principle Component Analysis (PCoA) of four *Ecklonia radiata* cohorts (*N* = 297), shown for five subsets of the SNP data; all loci (*n* = 1601), putatively neutral loci (*n* = 1559), all outlier loci (*n* = 42), provenance‐associated outlier loci (*n* = 27) and hatchery‐associated outlier loci (*n* = 15). (b) Pairwise *F*_ST_ estimates between corresponding cohorts, Values significant following the FDR correction shown in bold.

Genetic differentiation between warm and cool wild donor populations was relatively strong (pairwise *F*_ST_ across all SNPs = 0.083, pairwise *F*_ST_ for provenance‐associated SNPs = 0.356; Figure 2) and this differentiation was maintained and slightly elevated when comparing hatchery cohorts of contrasting provenance (*F*_ST_ across all SNPs = 0.092, *F*_ST_ provenance‐associated SNPs = 0.374). There was only a small amount of overall genetic differentiation between hatchery cohorts and their respective wild donors; this was mainly due to kelp of warm provenance for the overall dataset, but was accentuated in the hatchery‐associated loci dataset for both Warm and Cool hatchery cohorts (cool: *F*_ST_ across all SNPs = 0.001, *F*_st_ provenance‐associated SNPs = 0.01, *F*_ST_ hatchery‐associated SNPs = 0.064; warm: *F*_ST_ all SNPs = 0.015, *F*_st_ provenance‐associated SNPs = 0.024, *F*_ST_ hatchery‐associated SNPs = 0.116; Figure 2).

**FIGURE 3:**
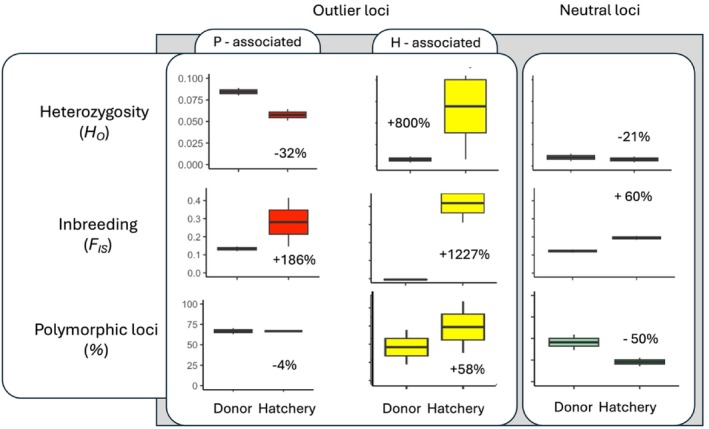
Genetic impacts of hatchery propagation on seaweed populations, showing changes in observed heterozygosity (*H*_o_), inbreeding coefficient (*F*_is_), and percent polymorphic loci (PL). Based on putatively neutral (*n* = 1559), provenance‐associated outliers (*n* = 27) and hatchery‐associated outliers (*n* = 15) loci sequenced across Donor (*n* = 199) and Hatchery (*n* = 102) *Ecklonia radiata* cohorts (pooled population diversity metrics across both warm and cool populations sampled at 5, 7 and 9 months old).

Cool donors exhibited roughly double the genetic diversity of warm donors (e.g., *H*_e_ neutral: 0.017 vs. 0.006). This pattern remained in hatchery cohorts but diversity declined modestly relative to donors, with neutral H_0_ decreasing by ~21% on average (Table 2, Figure 3).

Inbreeding coefficients (*F*_IS_) were positive and significantly different to zero across wild populations (> 0.17) and were modestly higher in hatchery kelp, particularly within the cool cohort (> 0.2; Table 2).

Neutral genetic diversity (*H*_o_ and AR) of hatchery cohorts was broadly similar to that of donors (Table 2; Figure 3). Metrics that captured rare alleles (PL and PA) indicated greater than 50% declines in neutral and provenance‐associated diversity in hatchery compared to wild cohorts (Table 2, Figure 3). In contrast, hatchery‐associated loci showed increased diversity, particularly in the warm cohort, which contained three fixed heterozygous alleles absent from both wild populations—strong evidence of hatchery‐driven selection or very early stochastic processes (Table 2, Figure 3). No consistent temporal trend was detected across 5‐, 7‐ and 9‐month timepoints (Table 2, Figure 3).

**FIGURE 4:**
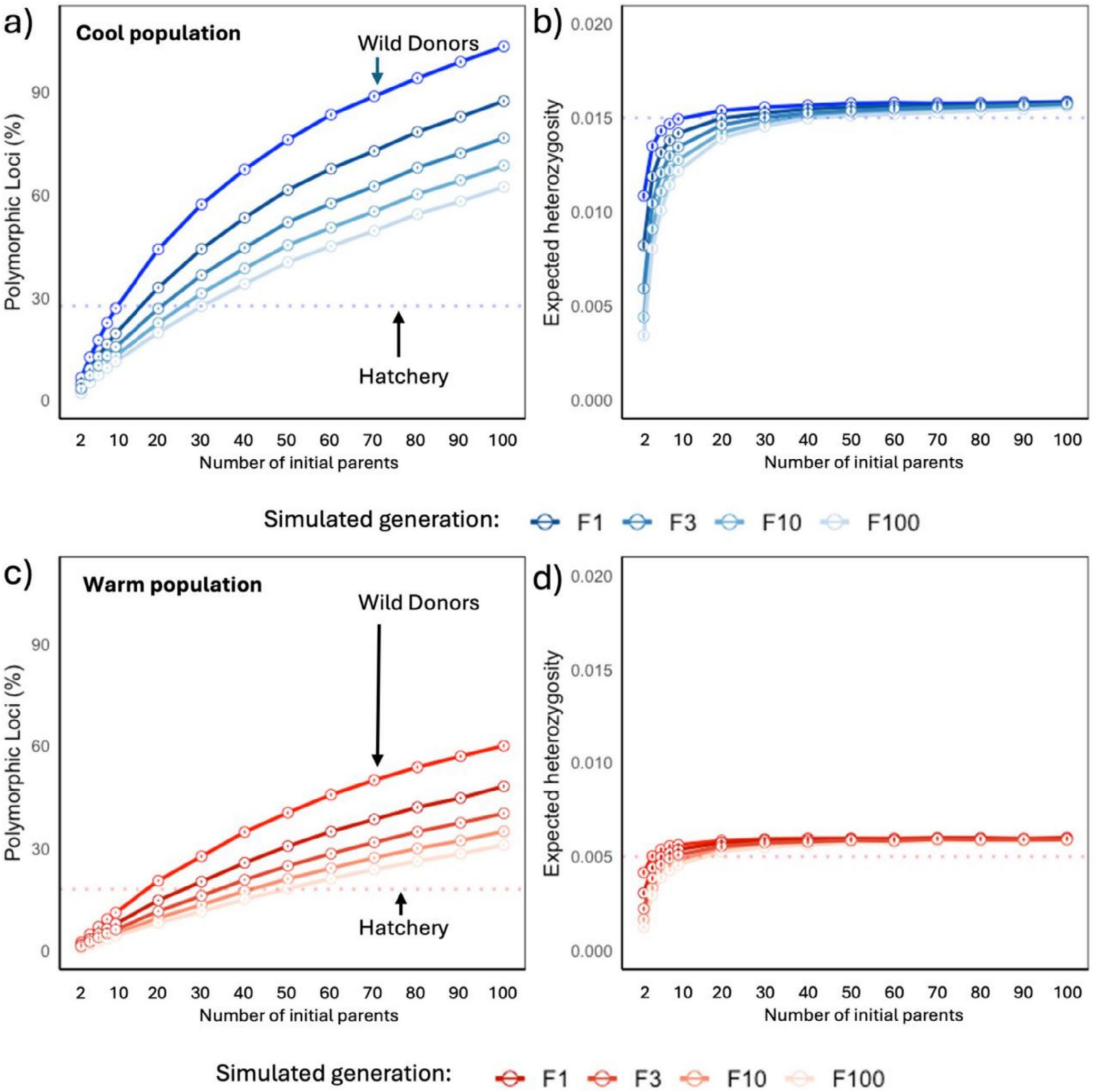
Impact of initial number of donor kelp on genetic diversity estimates for cultivated and restored populations, under best case scenarios. Mean ± standard error expected heterozygosity (left) and percentage of polymorphic loci (right) for simulated *F*_1_, *F*_3_, *F*_10_ and *F*_100_ offspring, calculated over 100 bootstrap replicates of 2–100 initial donors (*x*‐axis). Estimates were calculated across 1559 neutral SNPs from wild donor *Ecklonia radiata* from two populations in Western Australia. Mean values for expected heterozygosity and percentage of polymorphic loci for true (non‐simulated) *F*_1_ generations sampled in the hatchery indicated by red dotted lines.

Our simulations revealed that H_*E*_ plateaued by ~20 donors in *F*_1_, but maintaining this across *F*_100_ required ~100 donors. PL increased continuously with donor number, showing no saturation at 100 donors (Figure 4). Empirical warm *F*_1_ diversity corresponded to simulations with only ~4 donors under a random mating model, consistent with a genetic bottleneck. Observed *F*_1_ heterozygosity was consistently lower than predicted by the simulations (Figure 4), likely reflecting departures from model assumptions such as random mating and equal parental contribution, and indicating reduced realised genetic diversity in hatchery cohorts.

### 3.2 Survival and Growth

**FIGURE 5:**
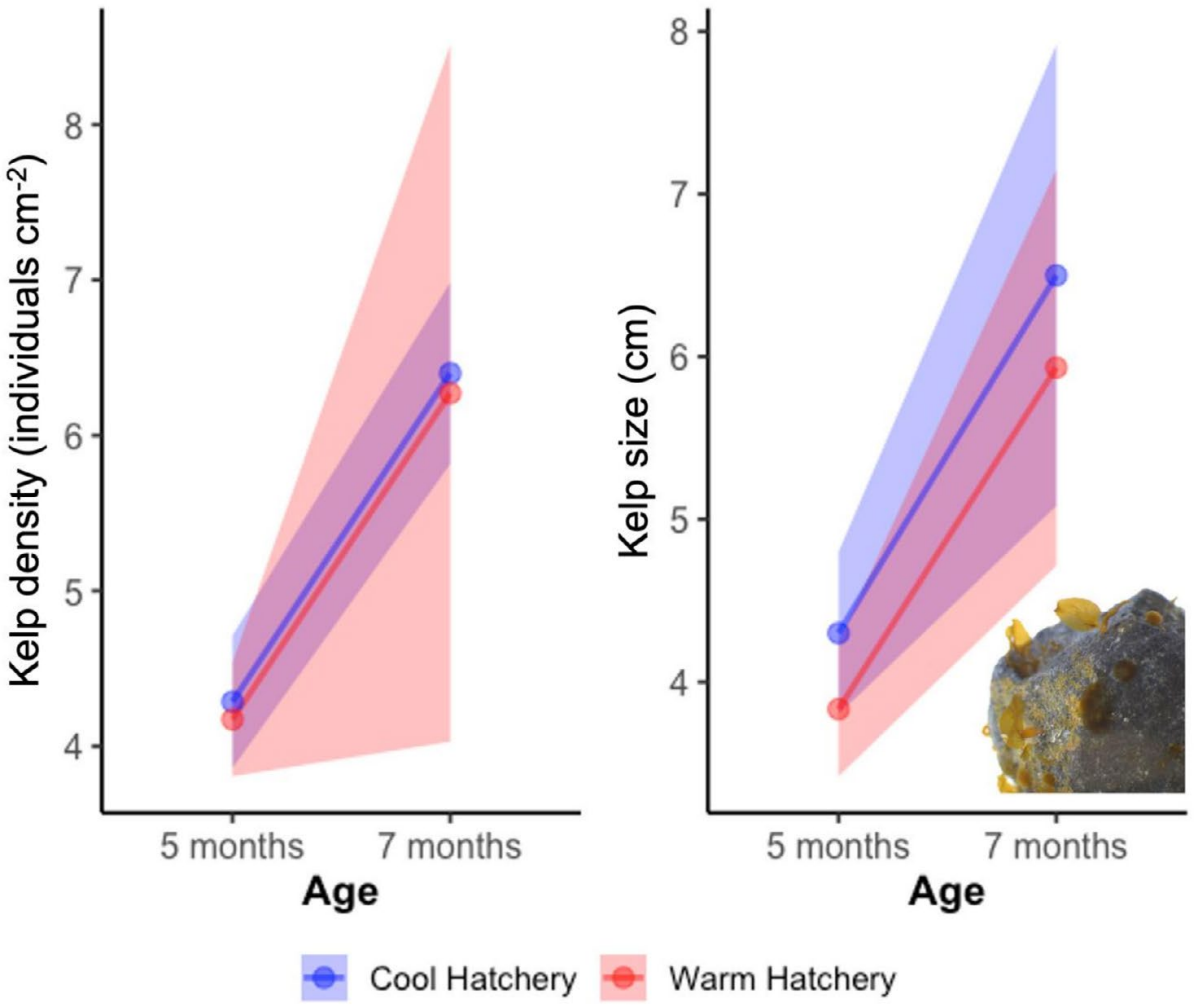
Density and growth of kelp in the hatchery. Effect of provenance (cool vs. warm) and month in hatchery (5 vs. 7) on density and size (mean ± s.e shown).

Both warm‐ and cool‐provenance kelps grew successfully in the hatchery (Figure 5, Table S2). Density increased slightly (4–6 cm^−2^) but not significantly (*F*_1,56_ = 0.51, *p* > 0.4). Size of sporophytes increased significantly with time (4–6 cm; *F*_1,56_ = 4.62, *p* = 0.04). No provenance effect or interaction was detected on density or size (*F*_1,56_ = 0.001, *p* > 0.9; *F*_1,56_ = 0.004, *p* > 0.9, respectively).

## 4 Discussion

**FIGURE 6:**
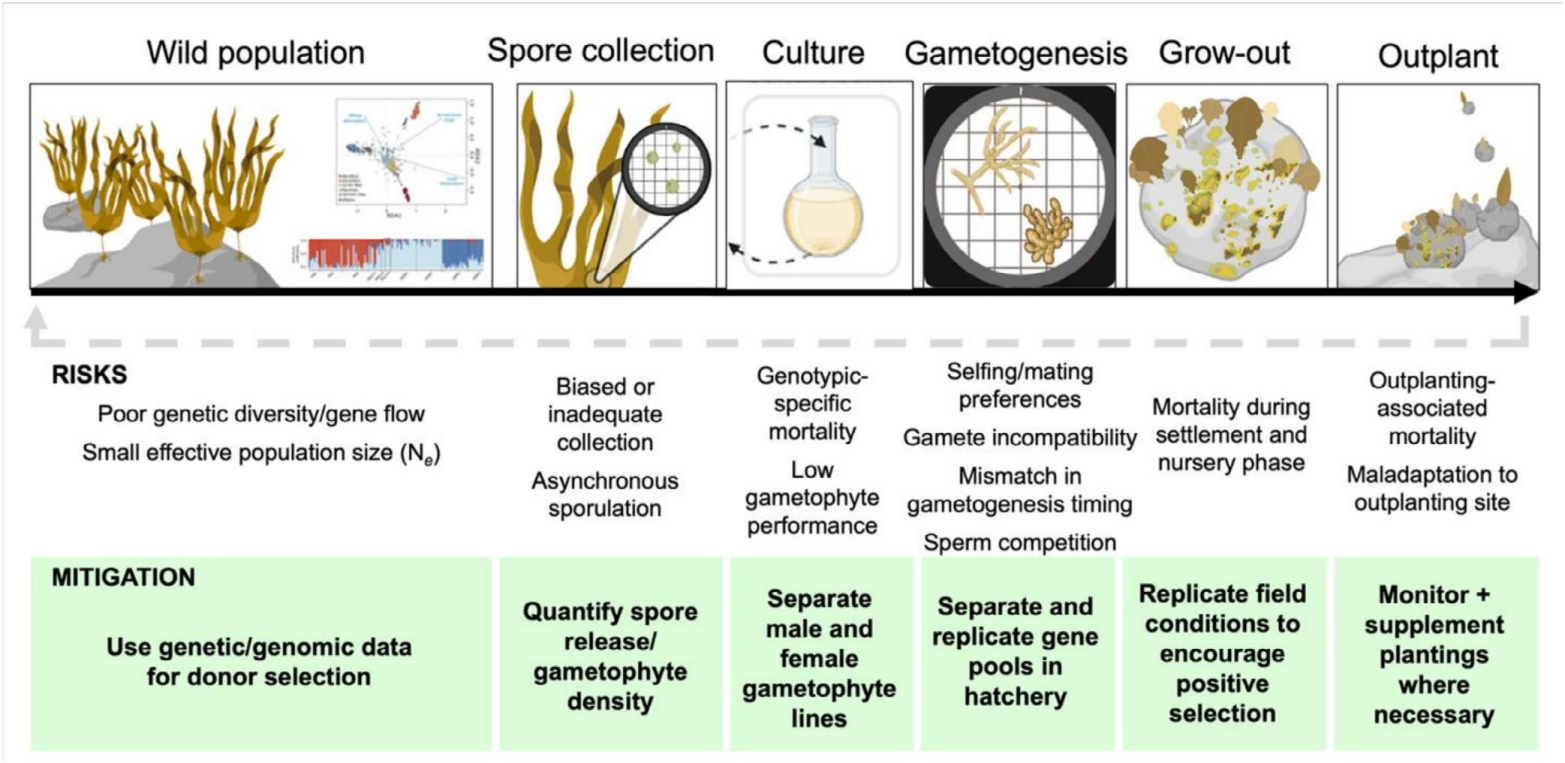
Conceptual framework illustrating genetic risks across the restoration pipeline and points for mitigation. Adapted from Baums (2008). Each step of the restoration workflow can alter the genetic composition of restored cohorts. Donor selection determines the initial breadth of diversity entering the pipeline, while variation in sorus size, spore release, and fertilisation success can create strong reproductive skew and elevate inbreeding. Early gametophyte development and nursery cultivation expose cohorts to controlled conditions that may favour particular genotypes, generating early domestication signals. Following outplanting, environmental filters and differential survival further modify allele frequencies. Key mitigation options include increasing donor numbers, standardising spore release, maintaining separate gametophyte lines or sexes to equalise parental contribution, aligning hatchery conditions more closely with field environments, and incorporating routine genetic monitoring to detect early drift or selection.

Aquaculture‐based restoration offers important opportunities to rebuild degraded marine habitats, yet our results show that even the earliest stages of propagation can substantially reshape the genetic composition of restored populations. A single hatchery generation of kelp was sufficient to generate strong genetic drift, elevated inbreeding and shifts in allele frequencies at loci associated with both provenance and hatchery conditions. Notably, these changes occurred despite high apparent performance in the hatchery, indicating that early cultivation success does not necessarily translate into the genetic characteristics required for long‐term restoration, and that evolutionary risks can arise during the initial stages of propagation. Here, we explore the mechanisms driving these genetic changes during propagation, consider their potential consequences for outplanting and persistence, and discuss their implications for restoration practice and policy (Figure 6).

### 4.1 Hatcheries Impose Powerful Evolutionary Filters

The reduction in effective population size (*N*_e_) from ~500 in the wild to 21–74 in the hatchery demonstrates that reproductive bottlenecks can emerge rapidly under mass‐spawning conditions. Only 21%–74% of donor individuals contributed genetically to the *F*_1_ generation, indicating that a substantial proportion of available genetic diversity—particularly rare alleles—was lost at the outset of propagation. This pattern aligns with observations from finfish, shellfish and coral hatcheries, where unequal parental contributions drive drift, reductions in heterozygosity and increased inbreeding (Araki et al. 2007; Christie et al. 2012). Such reproductive skew is therefore also likely to be a key mechanism shaping genetic outcomes in kelp restoration systems, unless measures are developed and taken to mitigate this risk (Baums 2008; Baums et al. 2022).

Elevated inbreeding coefficients, particularly within warm‐provenance cohorts, further suggest that kin mating and self‐fertilisation contributed to reduced genetic diversity. These processes are plausible in laminarian kelps, where gametophytes develop in close proximity and reproductive synchrony varies among individuals. Selfed or inbred sporophytes typically show reduced growth and competitive performance relative to outcrossed individuals (Raimondi et al. 2004; Johansson et al. 2013), and recent field trials indicate that selfed individuals are less likely to survive early outplanting stages (Dykman et al. 2025). As climatic variability intensifies, reduced genetic variation and elevated inbreeding may constrain the capacity of restored populations to persist under changing environmental conditions (Wernberg et al. 2018). Hence, mitigating this risk through ensuring more even parental contributions to outplanted generations is essential.

### 4.2 Early Domestication Responses Occur Alongside Drift and Inbreeding

Genomic analyses revealed two forms of genetic divergence: (i) persistent differences associated with donor provenance, and (ii) shifts in allele frequencies arising during the hatchery phase alone. The former likely reflect long‐term adaptation to regional environmental regimes, while the latter are consistent with changes occurring within a single generation under controlled conditions. Although most hatchery‐associated loci lacked functional annotation to the closest available reference genome, this may reflect phylogenetic divergence rather than a true absence of functional relevance. The consistent association of these loci with cultivated cohorts across tanks and rocks suggests that environmental conditions in the hatchery (e.g., temperature, light, flow, nutrient availability or microbial conditions) may have influenced allele frequencies. However, because tanks and substrates were inoculated from a common mixed spore slurry, we cannot exclude the possibility that early stochastic processes (e.g., variance in reproductive success or genetic drift during initial settlement) contributed to these patterns and were subsequently propagated during grow‐out. Together, these results are consistent with a combination of early‐stage stochasticity and potential selection under hatchery conditions.

### 4.3 Loss of Allelic Diversity Signals Vulnerability Despite Stable Heterozygosity

Genetic metrics also showed differing sensitivities to hatchery propagation. Expected heterozygosity changed little between donor and hatchery cohorts, but allelic richness, private alleles and the proportion of polymorphic loci declined substantially. These metrics are sensitive to the loss of rare variants that contribute disproportionately to adaptive capacity (Allendorf and Luikart 2006; Hoban et al. 2020). Recent whole‐genome data from the bull kelp (*Nereocystis luetkeana)* show that reductions in effective population size can rapidly elevate drift load and erode adaptive potential, even when heterozygosity appears relatively stable (Bemmels et al. 2025). Our forward simulations reinforce this pattern, as modest donor numbers were sufficient to maintain heterozygosity, but retaining rare alleles required substantially larger broodstock numbers. Monitoring heterozygosity alone may therefore mask early losses of evolutionary potential during hatchery propagation. As most kelp restoration programs use fewer than 30 donors (Wood et al. 2024; Dykman et al. 2025), erosion of adaptive variation is likely unless donor numbers are increased and breeding designs are optimised for the sector.

### 4.4 Genomic Composition May Help Explain Low Early Outplant Survival

The 9‐month samples analysed here were collected from individuals in the hatchery only; however, outplanting trials were conducted separately on a subset of individuals. Preliminary field observations indicated equally low persistence of outplanted sporophytes from both provenance groups (Figure S2). Although in situ environmental stressors undoubtedly contribute to early mortality in the field, the genetic changes observed here (reduced allelic diversity, elevated inbreeding and early domestication) provide plausible additional mechanisms for their loss. Similar mismatches between hatchery and field performance have been documented in finfish, shellfish and corals, where genotypes favoured in culture or hatchery environments perform poorly in natural environments (Naish and Hard 2008; Christie et al. 2014; Bosch et al. 2023). Outplant success depends not only on retaining overall genetic diversity, but also on minimising maladaptive selection during propagation. Hatchery environments that better reflect field conditions, including realistic temperature and light regimes, natural microbial communities, variable flow or staged stress conditioning, may help reduce unintended domestication and favour genotypes capable of persisting under natural selection.

### 4.5 Implications for Marine Restoration Design and Policy

Our results have direct implications for kelps and the marine restoration sector more broadly, as the sector enters a stage of heightened investment in large‐scale activities (Figure 6). First, donor pools need to be sufficiently large to retain rare alleles. Our empirical and simulation results suggest that > 100 donors may be required to achieve this in many cases. Second, modifying protocols for spore release and fertilisation to reduce reproductive skew can increase realised *N*_e_. This can be achieved by maintaining separate gametophyte family lines or male–female cultures and using controlled crosses to equalise contributions among donors. For example, our kelp restoration work in this area now separates hatchery tanks into multiple groups of 10 distinct individuals, to maximise genetic diversity retained. Third, aligning hatchery conditions with environmental regimes at restoration sites, through temperature matching, natural microbial communities, realistic flow regimes or shortened culture periods, may reduce domestication effects and ensure that restored populations are best adapted to field conditions. Finally, integrating routine genetic monitoring into restoration pipelines would allow practitioners to detect the risks of early drift, inbreeding and selection and adapt protocols accordingly.

### 4.6 Future Steps and Conclusion

Our results demonstrate that hatchery propagation can reshape the genetic composition of restoration cohorts within a single generation, even when donor numbers are high and short‐term performance appears strong. The reduction in rare alleles, elevated inbreeding and signals of early domestication observed here indicate that evolutionary risks can arise well before outplanting. As marine restoration scales globally, integrating genetic safeguards into propagation pipelines will be essential to ensure that restored populations retain the adaptive capacity required to persist under accelerating environmental change.

Genetic management must become a formal and enforceable component of marine restoration practice. Quantitative targets for donor number, realised effective population size and diversity retention should be embedded within project design, permitting frameworks and funding criteria. Without explicit standards and monitoring, restoration efforts risk unintentionally eroding the evolutionary resilience they seek to rebuild.

## Funding

G.W. was supported by an Australian Research Council (ARC) Linkage Grant (LP190100346) and an ARC Industry Fellowship (IE230100464). T.W., M.A.C. and K.F.‐D. were supported by ARC grants DP190100058 and LP190100346, KFD by ARC Fellowship FT230100214, and T.W. and M.A.C. by ARC grant DP240100230. T.W., K.F.‐D., G.W. and M.A.C. also received funding from the Norwegian Research Council (GecoKelp Project no. 335371), and T.W. and K.F.‐D. were also supported by the Schmidt Marine Technology Partners.

## Conflicts of Interest

The authors declare no conflicts of interest.

## Supporting information


**Table S1:** Genetic diversity metrics across time, including the breakdown of both sampling points at 7 months for the warm hatchery cohort.
**Figure S1:** RDA analysis plot showing loci in grey and cohort in coloured circles which match Figure 2 in the main text.
**Table S2:** ANOVA output on LMM testing the effects of provenance and age on kelp density and size in the hatchery.

**Figure S2:** Depicts low survival and growth of kelp in the hatchery versus field.

## Data Availability

Data for this study are available at the Dryad Digital Repository: https://doi.org/10.5061/dryad.2v6wwq049.
