# Supplementary material for: Minimising Risks of Reduced Genetic Diversity in Marine Restoration
Source: Evol Appl. 2026 May 20;19(5):e70257. doi: 10.1111/eva.70257 (PMC13239296; doi:10.1111/eva.70257)
Supplement: Supplementary file 1 — Table S1: Genetic diversity metrics across time, including the breakdown of both sampling points at 7 months for the warm hatchery cohort. Figure S1: RDA analysis plot showing loci in grey and cohort in coloured circles which match Figure 2 in the main text. Table S2: ANOVA output on LMM testing the effects of provenance and age on kelp density and size in the hatchery. [file EVA-19-e70257-s001.docx]

**Supplementary materials 1**

**Table S1. Genetic diversity metrics across time, including two sampling points at 7 months for the warm hatchery cohort.** Based on putatively neutral (n = 1559) and outlier loci associated with Provenance (n = 27) and Hatchery (n = 15), sequenced across Donor (n = 199) and Hatchery (n = 102) *Ecklonia radiata* cohorts. PL = polymorphic loci; AR = rarefied allelic richness; PA = private alleles; H_o_ = observed heterozygosity; H_e_ = expected heterozygosity; *F*_IS_ = inbreeding coefficient.7(a) and 7(b) were sampled 6 days apart, with the cool hatchery cohort sampled at the same time as warm 7(b).

| **Population** | **Cohort** | **Age (months)** | **n** | **Neutral loci** | | | | | **Provenance-associated outliers** | | | | | **Hatchery-associated outliers** | | | | |
| --- | --- | --- | --- | --- | --- | --- | --- | --- | --- | --- | --- | --- | --- | --- | --- | --- | --- | --- |
|  |  |  |  | **PL (%)** | ***PA (%)*** | ***Ho*** | ***HE*** | ***FIS*** | **PL (%)** | ***PA (%)*** | ***Ho*** | ***HE*** | ***FIS*** | **PL (%)** | ***PA (%)*** | ***Ho*** | ***HE*** | ***FIS*** |
| ***Warm*** | ***Wild*** | **>12** | **98** | **36.626** | **20.975** | **0.005** | **0.006** | **0.200** | **62.963** | **3.704** | **0.080** | **0.095** | **0.146** | **60.000** | **0.000** | **0.011** | **0.011** | **-0.005** |
|  | ***Hatchery*** |  | **62.00** | **17.960** | **3.784** | **0.004** | **0.006** | **0.183** | **66.667** | **0.000** | **0.051** | **0.640** | **0.150** | **93.333** | **20.000** | **0.127** | **0.171** | **0.323** |
|  |  | 5 | 12 | 29.787 | 0.449 | 0.003 | 0.005 | 0.250 | 18.519 | 0.000 | 0.045 | 0.044 | -0.003 | 73.333 | 0.000 | 0.130 | 0.221 | 0.360 |
|  |  | 7 (a) | 18 | 2.053 | 1.026 | 0.002 | 0.002 | 0.029 | 29.630 | 0.000 | 0.054 | 0.047 | -0.042 | 73.333 | 0.000 | 0.164 | 0.165 | 0.061 |
|  |  | 7 (b) | 15 | 4.745 | 0.641 | 0.005 | 0.006 | 0.359 | 44.444 | 0.000 | 0.067 | 0.083 | 0.153 | 60.000 | 0.000 | 0.077 | 0.096 | 0.164 |
|  |  | 9 | 17 | 7.953 | 1.155 | 0.004 | 0.006 | 0.366 | 29.630 | 0.000 | 0.037 | 0.059 | 0.245 | 80.000 | 0.000 | 0.112 | 0.156 | 0.248 |
| ***Cool*** | ***Wild*** | **>12** | **101** | **54.586** | **26.491** | **0.014** | **0.017** | **0.175** | **70.370** | **0.000** | **0.089** | **0.103** | **0.121** | **20.000** | **0.000** | **0.004** | **0.004** | **-0.005** |
|  | ***Hatchery*** |  | **36.00** | **27.646** | **3.784** | **0.011** | **0.016** | **0.206** | **66.667** | **0.000** | **0.064** | **0.111** | **0.415** | **33.333** | **0.000** | **0.008** | **0.028** | **0.526** |
|  |  | 5 | 9 | 12.957 | 1.347 | 0.015 | 0.019 | 0.226 | 48.148 | 0.000 | 0.097 | 0.122 | 0.200 | 13.333 | 0.000 | 0.008 | 0.033 | 0.807 |
|  |  | 7 | 18 | 16.164 | 1.411 | 0.009 | 0.015 | 0.361 | 55.556 | 0.000 | 0.054 | 0.093 | 0.348 | 6.667 | 0.000 | 0.004 | 0.017 | 0.773 |
|  |  | 9 | 9 | 7.633 | 1.090 | 0.008 | 0.012 | 0.315 | 51.852 | 0.000 | 0.051 | 0.112 | 0.440 | 26.667 | 0.000 | 0.015 | 0.040 | 0.500 |


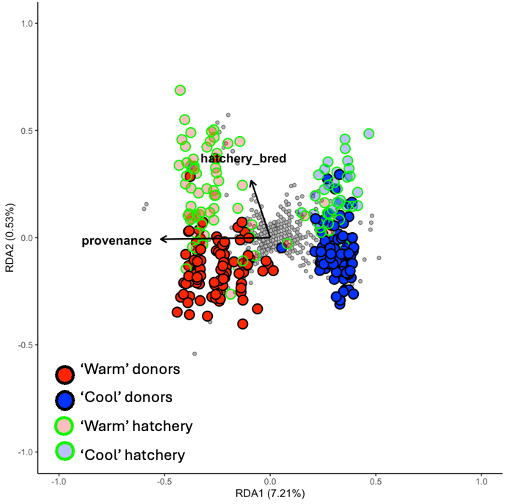


**Figure S1.** RDA analysis plot showing loci in grey and cohort in coloured circles.

**Table S2.** ANOVA output on LMM testing the effects of provenance and age on kelp density and size in the hatchery.

| Response | Effect | df | F | p-value | Significance |
| --- | --- | --- | --- | --- | --- |
| Density | Provenance | 1 | 0.0005 | 0.98 |  |
|  | Month (5 vs 7) | 1 | 1.63 | 0.21 |  |
|  | Provenance x Month | 1 | 0.004 | 0.95 |  |
| Size | Provenance | 1 | 0.51 | 0.48 |  |
|  | Month (5 vs 7) | 1 | 4.62 | 0.04 | ** |
|  | Provenance x Month | 1 | 0 | 0.97 |  |
