# Supplementary material for: Minimising Risks of Reduced Genetic Diversity in Marine Restoration
Source: Evol Appl. 2026 May 20;19(5):e70257. doi: 10.1111/eva.70257 (PMC13239296; doi:10.1111/eva.70257)
Supplement: Supplementary file 2 — Figure S2: Depicts low survival and growth of kelp in the hatchery versus field. [file EVA-19-e70257-s002.docx]

**Supplementary materials 2**

*Outplanting*

108 rocks with hatchery-bred sporophytes were transported ~650 km north in the austral spring to Kalbarri, Western Australia (*Ecklonia radiata*’s former northern range edge prior to widespread loss in 2011; Wernberg et al., 2016). A random subset of rocks from each tank (N = 50) was retained in the hatchery as a control treatment. Sporophytes were transported in a refrigerated truck cooled to 12 °C (18-hour transit) and outplanted via SCUBA to three sites located 2 km apart over a six-hour period. At each site, four plots of nine rocks placed in a 1-m radius circle were established ~10–20 m apart, and each plot was randomly assigned one provenance treatment (two plots of each provenance per site) (Fig. 1B).

Kelp sporophytes were outplanted at six months post-seeding. Divers subsequently returned to all sites at seven and nine months post-seeding to match the age of hatchery controls. During each SCUBA survey, sporophyte survival, attachment, and canopy size were recorded within each plot to assess early persistence and provenance-specific performance under field conditions.


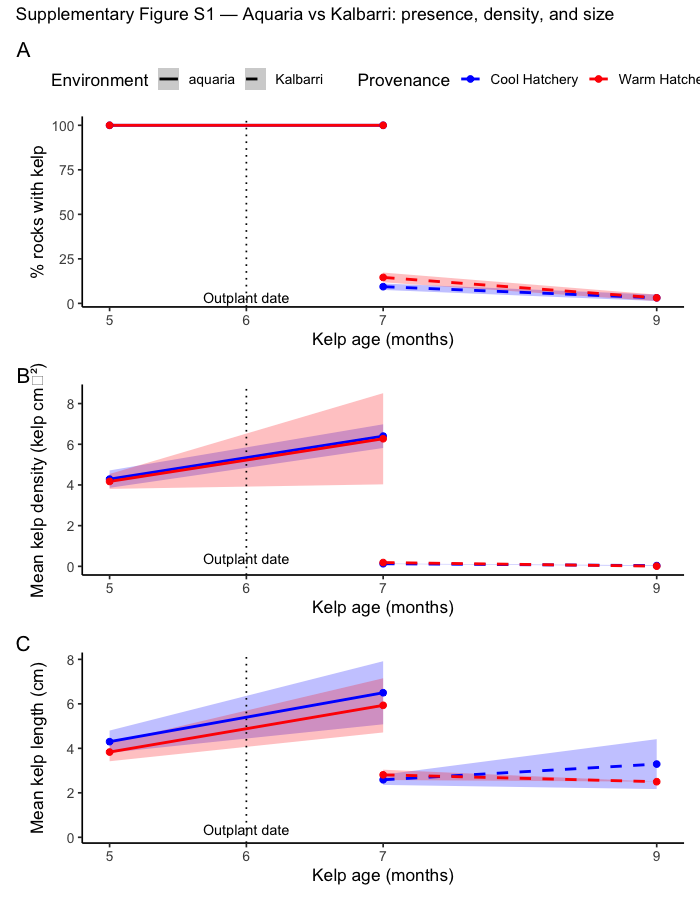


**Figure S2**. Survival and growth of kelp in the hatchery versus field.
